# Risk factors associated with road traffic injuries at the prone-areas in Kampala city: a retrospective cross-sectional study

**DOI:** 10.5249/jivr.v13i1.1347

**Published:** 2021-01

**Authors:** Joseph Kimuli Balikuddembe, Ali Ardalan, Kasiima M. Stephen, Owais Raza, Davoud Khorasani-Zavareh

**Affiliations:** ^*a*^ Institute for Disaster Management and Reconstruction, Sichuan University, Chengdu, China and Hong Kong Polytechnic University.; ^*b*^ Department of Disaster Public Health, School of Public Health, Tehran University of Medical Sciences, Tehran, Iran; T. H. Chan Harvard School of Public Health, Cambridge, USA.; ^*c*^ Directorate of Road Traffic and Road Safety, Uganda Police Force, Kampala, Uganda.; ^*d*^ President’s Primary Healthcare Initiative (PPHI) Sindh, Karachi, Pakistan.; ^*e*^ Department of Health in Emergencies and Disasters, School of Public Health and Safety, Shahid Beheshti University of Medical Sciences, Tehran, Iran.

**Keywords:** Road traffic injuries, Risk, Prone-area, Kampala, Uganda

## Abstract

**Background:**

Road traffic injuries (RTIs) pose a disproportionate public health burden in the low and middle-income countries (LMICs) like Uganda, with 85% of all the fatalities and 90% of all disability-adjusted life years lost reported worldwide. Of all RTIs which are recorded in Uganda, 50% of cases happen in Kampala —the capital city of Uganda and the nearby cities. Identifying the RTI prone-areas and their associated risk factors can help to inform road safety and prevention measures aimed at reducing RTIs, particularly in emerging cities such as Kampala.

**Methods:**

This study was based on a retrospective cross-sectional design to analyze a five year (2011 – 2015) traffic crash data of the Uganda Police Force.

**Results:**

Accordingly, 60 RTI prone-areas were identified to exist across the Kampala. They were ranked as low and high risk areas; 41 and 19, respectively and with the majority of the latter based in the main city center. The bivariate analysis showed a significant association between identified prone-areas and population flow (OR: 4.89, P–value: 0.01) and traffic flow time (OR: 9.06, P–value: 0.01). On the other hand, the multivariate regression analysis only showed traffic flow time as the significant predictor (OR: 6.27, P–value: 0.02) at identified RTI prone-areas.

**Conclusions:**

The measures devised to mitigate RTI in an emerging city like Kampala should study thoroughly the patterns of traffic and population flow to help to optimize the use of available resources for effective road safety planning, injury prevention and sustainable transport systems.

## Introduction

Worldwide, more than 1.3 million lives are lost each year as a result of road traffic injuries (RTIs). An estimated 3,700 people die on the world’s roads every day.^1^ However, a disproportionate burden of RTIs is heavily borne by the low and middle-income countries (LMICs) —where 85% of all the fatalities and 90% of all disability-adjusted life years lost worldwide are reported to occur. According to the latest world report on road safety, the rates of road traffic deaths are highest in Africa and South-East Asia with 26.6/100,000 and 20.7/100,000 people, respectively.^1^ RTIs are the leading cause of death among the young people aged 15-29 years and they cost governments, particularly in LMICs economic loss of between 1% and 3% of their Gross Domestic Product.^2-4^ This not only poses a huge public health burden to them but also hampers their economic development. RTIs are presented as the ninth leading cause of death and based on their upward trend, they are predicted to become the fifth leading cause of death by 2030 in LMICs unless urgent and appropriate actions are taken.^2,5,6^

RTIs are random events which occur disproportionately at specific locations — under the influence of different spatial patterns.^7-9^ Through risk mapping, both the safest and most dangerous road sections that are prone or experience abnormally high number of RTIs can be identified in a given region or country.^10-12^ It is important, however, to note that RTI prone-areas can vary from country to country depending on the severity and frequency of road crashes they experience. In this regard, there is still no universal definition of what qualifies to be called a RTI prone-area or hotspot.^10,13-15^ In Belgium, for example, the most dangerous crash sites for RTIs were determined by using the Analysis Form for Traffic Accidents. As a result, the locations, which had witnessed “3” or more crashes in the last three years, were henceforth selected to be a dangerous crash site. ^10^ Yet in another study conducted in Addis Ababa, Ethiopia; instead, the RTI black-spots were determined according to the ranking order and this was based on their frequencies of occurrences. The first 10 hazardous sites in the ranking were thus, considered to be black-spots.^14^ The present study, however, herein uses “RTI prone-areas” other than hotspots or black spots. Ideally, identifying the RTI prone-areas can help in understanding and comparing their associated risk factors, spatial patterns, and discerning their relationship with the ongoing activities. Consequently, this can help to provide the policymakers with useful insights and information when deciding on the best remedial decisions for preventing and reducing the burden of RTIs, and particularly at locations where they happen on a regular basis.

The burden of RTIs is one of the critical public health challenges of the 21st century affecting many emerging cities in LMICs like Kampala —the capital of Uganda, which over recent years has been reported as one of the fastest-growing African cities, so far at a growth rate of 5.6%.^16-19^ Kampala, however, is still faced with several challenges including among others, poor physical planning, poor roads, rural-urban migration and overcrowding due to unprecedented population growth.^17,19,20^ These are also blighted with the motorized and non-motorized means of transport, which oftentimes use the same road networks with the vulnerable and unprotected road users —as it is a common problem witnessed in many cities of LMICs.^3,4^ It is undeniable that these challenges not only have an impact on Kampala’s urbanization and transport systems, but also can gradually exacerbate the underlying causes of RTIs. Accordingly, it is estimated that 27.4 per 100,000 population in Uganda die in RTIs annually.^2,21,22^ In the recent years of all RTIs reported in Uganda, ~50% cases and more were reported to have happened in Kampala and the nearby cities, particularly those which form the Kampala Metropolitan Area (KMA).^22-24^ More so, some of these incidents were reported to have continuously been occurring at same locations compared to others in KMA and elsewhere. However, it is worth highlighting that there is no study to-date explaining the possible reasons for the predominance of RTI in KMA as well as their risk factors in order to help to inform the decision making and interventions for effective road safety vis-à-vis injury prevention.^25^ This is why our study was undertaken to identify the RTI prone-areas and their associated risk factors in Kampala city based on a retrospective analysis of crash data for five years (2011-2015). 

## Materials and Methods

**Study Design**

A retrospective cross-sectional design was used to analyze the traffic crash data from the Uganda Police Force (UPF) for five years (2011 – 2015). A retrospective analysis combined with the cross-sectional design was employed because it is appropriate and routinely used to collect data from either the entire population, record collection/ and database or their subset thereof selected and from this, data is collected to help to answer the key research questions of interest. In the present study, this involved identifying the RTI prone-areas (at the road locations) and their associated risk factors in Kampala city and followed by visualizing their distribution across the 5 municipalities of Kampala with help of ArcGIS 9.3.

**Study area**

**Figure 1:**
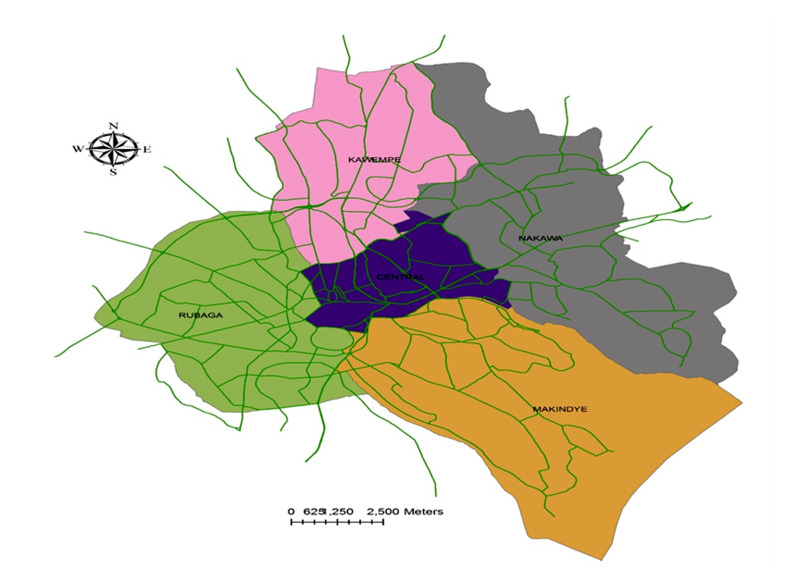
The five Municipalities of Kampala city (Prepared with GIS).

Kampala city covers a surface area of 189 km^2^ and its astronomical location is at latitude 0.3475964 and longitude 32.5825197. It is currently administered based on five (5) municipalities: Central (Cen), Kawempe (Kaw), Makindye (Mak), Nakawa (Nak) and Rubaga (Rub) (Figure 1). ^17,20^ The city is not only a home of 1.75 million people but also a working environment for a population estimated at 4.5 million and noted to grow at 4.5% per annum compared to the national average population growth of 3.03 per annum.^17-19^ Also, Kampala is the most developed and busiest area in Uganda with the major highways linking to the eastern, western and northern regions of Uganda as well as to the neighbouring countries (The Democratic Republic of Congo, Kenya, Rwanda, South Sudan and Tanzania). Of the approximated 1,200 km^2^ of roads in Kampala, however, only about 300 km^2^ (25%) are paved and tarred. It is worth noting that, at the present of all the motor vehicles registered in Uganda (approximately 800,000), 400,000 (60%) of them use Kampala roads yet its road network amounts to only 0.083% of all the estimated road length in the country.^17,18,20,26^

**Data source and data collection**

Between May 2015 and May 2016, the traffic crash data in the registries of the Directorate of Traffic and Road Safety of UPF between 2011 and 2015 were retrospectively extracted, reviewed, and analyzed with an aim of identifying the high RTI areas. However, some challenges involving the incomplete, inadequate or unavailable data on certain incidents were encountered. For the present study, this can be attributed to that fact that Kampala city in particular and Uganda at large have no formalized database for RTIs. Instead, related data are recorded in the traffic registries which only capture the variables including the crash date, time, scene/road location, nature or type, cause, victims, vehicles, and impact on each specific RTI.

Data from the seven (7) traffic departments at the UPF Division Headquarters in Kampala were randomly sampled on the 4-months basis for each year. In this case, the aforementioned variables for each particular case of RTI registered were extracted, reviewed and analyzed. Also, the Shapefiles (data layers), which were relaying the type, name, and location of roads across the five municipalities of Kampala city were acquired from the Department of Physical Planning of the Kampala Capital City Authority (KCCA).

The period between 2011 and 2015 for data acquisition was considered for this study. It because is when various road safety and injury prevention measures were reinforced in-line with the United Nations Decade of Action for Road Safety 2011- 2020 globally as well as in Kampala and Uganda in general as a signatory —with an ultimate goal to stabilize and reduce the forecasted level of road traffic fatalities worldwide.^27^

**Analysis and identification of RTI prone-areas in Kampala city**

According to the International Classification of Diseases and Causes of Death (ICD 10), traffic accidents are classified under the V01–V99 codes with specifications on fatality and injuries in the motor vehicle-related crash.^28^ Based on this, the present study considered the cases or events which were registered with the traffic departments in KMA to have involved a crash of vehicle with a motorized and non-motorized means, people, property or animal. RTIs were randomly reviewed on a 4-months basis for each year and entered in the Microsoft Excel spreadsheet 2010 according to four entries: crash date, scene/ or location, nature and impact in order to facilitate subsequent study analysis. Since there were neither specific months nor seasons as to when Kampala city in particular and Uganda at large witness a spike of traffic crashes, four months were therefore randomly considered in each year. This also aimed at achieving the representative numbers of RTIs in each year across the 7 traffic departments at the UPF Division Headquarters in Kampala.

Much as the GIS-related models and particularly kernel density estimation (KDE) is commonly used to determine or evaluate the ranking of RTI prone-areas —whether of low or high risk frequency, many other different methodologies have been also used but without any standardized criteria. Moreover as earlier noted with the cases of Belgium and Addis Ababa.^10,14^ RTI prone-areas can vary from country to country depending on the severity and frequency of road crashes they experience. In regard to this, a road location that was registered to have had ≥5 fatal or serious crash incidents were thus, identified to be a “RTI prone-area” and was then ranked as either low or high if it experienced between ≥5 and 10, and ≥11 RTIs, respectively. 

Based on WHO categorization of RTIs, herein, a fatal incident refers to the death/ or loss of life of a victims following a RTI while a serious incident is a catastrophic injury with significant or long-term impact on a victim and his/ her family after being involved in a traffic crash accident.^6^ Apart from the risk of sustaining permanent disability, the victims of traffic crashes who are oftentimes involved in either serious or fatal crash incidents barely survive, especially in LMICs like Uganda having weak healthcare and emergency systems to render timely responses to them. This, therefore, calls for the concerted road safety and preventive measures to reduce the avoidable fatalities, injuries, trauma and disabilities associated with RTIs. Against this reason, the present study chose to focus on identifying the locations in Kampala city which are prone to both fatal and serious RTIs.

Each identified RTI prone-area was entered in the Statistical Package for the Social Sciences (SPSS) version 22, with its information related to at least five key variables affecting the political and socioeconomic dynamics of Kampala city, including the geographical terrain, type of road infrastructure, time of traffic flow, population flows and ongoing human activities. The details of these variables are presented in Table 1. Both bivariate and multivariate logistic regression analyses were applied to establish the underlying relationship between the identified RTI prone-areas and above 5 variables at a significance level of 0.05.

**Table 1:** Details about the five variables which were considered for RTI prone-areas.

Variable	Attributes	Definition/particulars
Geographical terrain	Low-lying area	Area close to water source, wetland or swamp
	Flat area	Plain land area
	Sloping area	One point of an area is at higher level than the other
Road infrastructure	Paved	Surface covered with bituminous asphalt concrete
	Intersection	Road point where 2 or more roads meet each other
Time of the traffic flow	Morning and evening hours	Large traffic production, attraction and transfer mainly in the morning and evening peak hours (≥ 5,000 average traffic vehicles per hour)
	Continuous	Constant traffic production, attraction and transfer throughout the day ( ≥ 5,000 average traffic vehicles per 24 hours)
	Moderate	1,000 – 10,000 population per km^2^
	High	≥ 10,000 population per km^2^
Ongoing human activities	Socioeconomic	Trade, business, transport, housing, employment, markets, industry, manufacturing, construction, shopping, tourism, sports, leisure and entertainment etc.
	Socioeconomic and institutional	Government and non-government office related activities (Presidential, Ministries, Parliament, Judiciary, education and others) in conjunction with various socioeconomic activities

**Mapping the roads with RTI crash prone-areas in Kampala city using ArcGIS 9.3**

**Figure 2:**
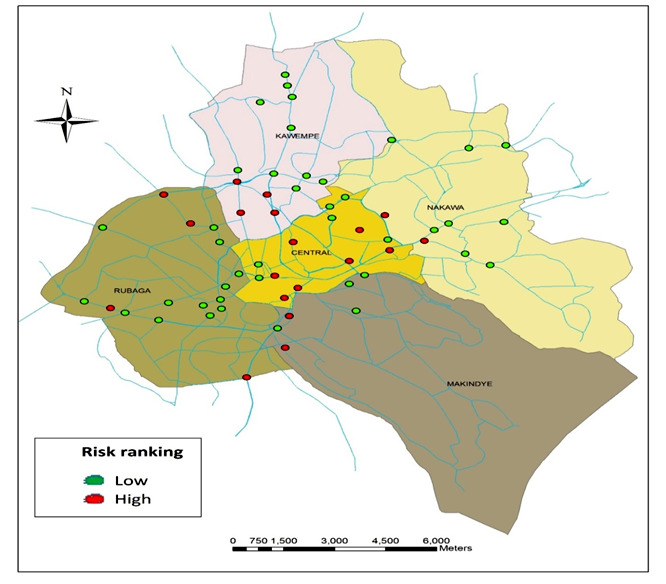
Categorization of road traffic crash prone-areas across 5 municipalities of Kampala city.

Layers along with the attributes of roads and municipalities across Kampala were exported, geocoded and georeferenced by using the different ArcGIS 9.3 symbology tools. Road locations which were identified to be prone to RTIs in each municipality were plotted using the polygon feature of the ArcGIS 9.3 software (Figure 2).

***Data sources: ***MoWT, 2010; JICA, 2010; UNRA, 2015; and KCCA, 2016

**Ethics clearance and approval**

Formal requests for the permission to obtain, access and use their data were submitted and granted both by the Directorate of Traffic and Road Safety of UPF, and the Department of Physical Planning of KCCA.^29,30^

## Results

In overall, 60 RTI prone-areas were identified and distributed across the 5 municipalities of Kampala city as follows: Central 13 (21.6%), Kawempe 15 (25%), Makindye 6 (10%), Nakawa 9 (15%) and Rubaga 17 (28.3%). The identified prone-areas for RTIs are visualized in figure 2 and ranked as high and low; 19 (31.6%) and 41 (68.3%), respectively.

The high-risk crash prone-areas were respectively distributed among the 5 municipalities as follows: Central 7 (36.8%), Kawempe 4 (21%), Makindye 2 (10.5%), Nakawa 2 (10.5%) and Rubaga 4 (21%). On the other hand, the low-risk RTI prone areas are distributed across 5 municipalities as follows: 6 (14.6%); 11 (26.8%); 4 (9.7%); 7 (17%); and 13 (31.7%) respectively. In this case, the high-risk RTI prone-areas were more predominant in the Central Municipality 7 (36.8%).

Based on Table 2, most of the identified crash prone-areas (33) their frequencies of traffic flows happened mostly during the morning and evening peak hours compared to others (27) whose traffic flow was always continuous. However, the time of traffic flow at the high-risk RTI prone-areas (78.5%) was continuous, especially during the weekly working days.

**Table 2:** Bivariate and multivariate analysis showing the risk association between ranking of crash areas and five variables in Kampala city.

Variable	Risk ranking of prone-areas		Bivariate analysis				Multivariate analysis			
	Low	High	OR	95% CI		p-value	OR	95% CI		p-value
	%	%		LL	UL			LL	UL	
**Geographical terrain**										
Low-lying	5 (12.2)	5 (26.3)	3.00	0.61	14.86	0.18	1.03	0.15	6.91	0.93
Flat	21 (51.2)	9 (47.4)	1.29	0.36	4.62	0.70	1.02	0.23	4.36	0.98
Sloping	15 (36.6)	5 (26.3)		Reference			Reference			
**Road infrastructure**										
Paved	30 (73.2)	11 (57.9)	Reference				Reference			
Intersection	11 (26.8)	8 (42.1)	1.98	0.63	6.22	0.24	0.57	0.13	2.57	0.46
**Population flow**										
Moderate	32 (78.1)	8 (42.1)	Reference				Reference			
High	9 (21.9)	11 (57.9)	4.89	1.51	15.80	0.01	3.03	0.55	16.70	0.20
**Traffic flow time**										
Morning and evening	29 (70.7)	4 (21.1)	Reference				Reference			
Continuous	12 (29.3)	15 (78.5)	9.06	2.49	32.98	<0.01	6.27	1.31	29.89	0.02
**Human activities**										
Socioeconomic	26 (63.4)	10 (52.6)	Reference				Reference			
Socioeconomic and institutional	15 (36.6)	9 (47.4)	1.56	0.52	4.70	0.43	2.20	0.51	9.51	0.29
Constant						0.09				<0.01
**Multivariate model summary**										
Nagelkerke R^2^								0.32		
% of correct prediction								73.3		

The population flows at most of the RTI prone-scenes (40) were moderate compared to other identified prone-areas (20) —which instead showed to experience the busy population flows. Eleven (11) high-risk crash areas (57.9%) witnessed the high population flows.

The bivariate analysis identified only two (2) out of the five (5) variables to be significantly associated with the RTI prone-areas, which mainly included the population and time of traffic flows (see table 2). Also, table 2 shows the adjusted odds ratio (aORs) and their associated 95% confidence interval (CI). After adjusting the influence or impact of the geographical terrain, type of road infrastructure, population flow and the ongoing human activities; the time of traffic flows was identified to have remained statistically significant. This, therefore, reflected that when the traffic flow is continuously heavy so as the RTI prone-areas were more likely to be exposed to a high risk of RTIs (OR: 6.27, P-value: 0.02).

## Discussion

This study labored to identify as well as to visualize the identified RTI prone-areas and their underlying risk factors across the five municipalities of Kampala city, which is one of the emerging and fastest-growing cities in Africa.^16,19^ It revealed both the traffic time and population flows as the most significant (based on their P-values of <0.01&0.02 and <0.01 &0.20, respectively) underlying risk factors to influence the occurrences of RTIs in Kampala city. In this case, the two patterns reflect the exponential traffic and transient population flows in Kampala, especially in the Central Municipality, consisting of the Kampala Central Business District (KCBD) —which is so far the busiest area with Kampala city. The two main findings not only highlight the potential risk factors of RTIs but also offer an opportunity to evaluate the existing traffic standards and services that are needed for both the motorized and non-motorized road users who are reported to flow in large numbers to the different parts of Kampala city, and particularly to KCBD. On the other hand, the findings can be relied on in informing the injury prevention measures aimed at reducing the occurrence and impact of RTIs.

Unsurprisingly, both the high traffic and population patterns in Kampala city could be attributed to its status of being not only the political epicenter but also an economic hub of Uganda —so far accounting for 80% of the country’s industrial and commercial activities.^17^ This corroborates a similar situation of many other emerging cities elsewhere, for example in China, India, Indonesia, Nigeria, South Africa, Turkey, and Vietnam where large population flows were reported to have impacted on their urban structures and motorization.^31^ Accordingly, an estimate of over 3 million and 1 million population daily flows to Kampala during the day and night hours, respectively.^17,20,26^ Similarly, over 42.4% commercial motorcycles, 36.6% motor cars and 21% light omnibus also daily flow to Kampala.^32^

In regard to the above, the concern is whether the existing road networks are sufficient enough to handle such patterns of traffic and population flows in this emerging African city. It is undeniable that a situation of this nature is likely to be placing a tremendous burden towards the only ~1200 km^2^ paved road network found in Kampala city, which was designed and constructed to cater for only 100,000 vehicles. Yet to date, the rapidly increasing traffic of over 60% of all the total registered vehicles in Uganda is reported to be concentrated in Kampala.^17,32^ A potential risk that can be created by this situation is of the pedestrian and vehicular conflicts, whereby different road users interact or use the same streets or roads with both the motorized and non-motorized means as it is a common problem faced in most of LMICs.^3,4^ More so, the heavy traffic and population flows can overwhelm the traffic enforcement capacity and also lead to quick wear and tear of roads and subsequently their dilapidation —which challenges were also previously cited to be related in one way another to the risk of RTIs in Kampala. ^17,20,22,32,33^

Considering this study’s findings, a shift of Kampala’s transport systems that is “people-and-not-car-oriented” is preferably and urgently needed, and could be one of the most appropriate mechanisms to address the risk factors for RTIs emanating from the large and unregulated traffic flows in Kampala. This should along be implemented by regulating the number of commuter 14-seater light omnibuses (commonly known as Kamunyes) and the commercial motorcycles (known as Boda-bodas) which are ubiquitous all over Kampala and have been contributing to a high proportion of traffic in the city for over years.^20,32-34^ Already quite a similar approach was successfully implemented in Kigali - Rwanda and as a result, it significantly helped to lead to substantial reductions in RTI-related mortalities which were attributed to the motorcycles.^35^ This is thus worth emulating for Kampala city and Uganda as a whole —where the risk of and an alarming burden of RTIs is mostly associated with the commercial motorized of transport means, and particularly the Boda Bodas and Kamunyes.^17,21-24,36^

To overcome the challenges of rapid traffic and population flows in Kampala which also contributed to both unregulated and mixed traffic,^21,33^ it is necessary to assign the specific travel time to some types of vehicles. This can be basically implemented for the vehicle traffic flows during the day and nighttime as well as over the weekend (Saturday and Sunday). In this case, the night hours should preferably be assigned to the heavy-goods-vehicles such as the lorries or trailers, unlike the commuter vehicles whose transportation services at most are need to run almost throughout. In the settings where there’s still inadequate capacity to enforce the traffic laws, however, some precautions need to be taken before regulating or implementing any travel time limitations for vehicles. This is because addressing issues of traffic management, enforcement, public transport regulation, and other related challenges requires a significant professional capacity.^31^ For instance, the habits related to drinking and driving and the use of drugs are oftentimes prevalent among night and weekend drivers as is commonly witnessed in the United States.^37^ Moreover, over the years the bad habits of drunk driving and drug abuse in some drivers and motorcyclists have been reported as among the serious causes of RTIs in Kampala and elsewhere in Uganda. Despite this, the country is still facing the challenge of inadequate personnel and resources to tackle these pressing issues.^22,34,36^

Also, the findings in this study underscore the overarching role of the pedestrian and traffic calming facilities, especially at the locations which experience heavy traffic flows and pedestrian movements. Traffic and pedestrian calming facilities needed include pedestrian walkways, overpasses, demarcated bus stops, footbridges, crosswalks, speed limits, lane markings, roadside barriers, speed humps, and warning and danger signs.^2-4,38^ These facilities are useful and can augment road safety measures aimed at regulating the traffic flows and road users’ behaviors such as street crossing, limiting speeds, a pedestrian walking and alerting road users of any potential hazards. They are also vital in regulating the mixed, disorderly or congested traffic which can ultimately help in reducing RTIs.^39,40^ Their inadequacy or absence, in one way another contribute to the negative behaviors of road users which in-turn aggravate some of the risk factors of RTIs like mixed traffic at certain locations, especially those known to attract high population flows, for example, the business and trade centers, markets, shopping malls and so on. A related challenge in this regard was reported in Adelaide Metropolitan Area, in Australia; whereby the absence of some of the traffic facilities was identified as one of the major causes of pedestrian-vehicle crashes and unsafe bus stops. This was particularly a challenge identified at some of the locations that experienced the high pedestrian turning movements.^11^ Therefore, the ongoing infrastructural planning, development and road upgrading in Kampala city,^17,19,20,26^ ought to incorporate the traffic engineering measures, especially those focusing on the vital pedestrian and traffic schemes. This should also strive to prioritize the pedestrian walking facilities since a large percentage of road users in emerging cities like Kampala and elsewhere in many LMICs mostly rely on walking or cycling and also commute by public transport.^2-4^

**Strength and limitation**

To the best of our knowledge, there’s a paucity of official information on the RTI prone-areas and their associated risks in Kampala city and also in many other similar settings, a gap this study in one way another tried to bridge. However, given that there’s no agreed definition of a RTI prone-area nor that for a hotspot; the study concentrated on identifying the roads which witnessed more than 5 fatal or serious RTIs between 2011 and 2015. To this matter, some of the roads that are exposed to a higher level of risk to RTIs but did not experience either any fatal or serious RTIs within the study period could have been left out. Besides, as aforementioned, we encountered some challenges related to the incomplete, inadequate, missing or unavailable data —which made it quite hard for us to conduct a more detailed study showing the total numbers of RTIs at each prone-area. Problems related to data were not only exceptional to our study since it has been previously reported as among the challenges hindering the promotion of road safety in other developing countries such as in Rwanda and in East Asia.^7,35^

## Conclusion

The identified RTI prone-areas and their associated risk factors in Kampala city were noted to be more influenced by the traffic time and population flows. Therefore, the measures which are devised to remedy them should thoroughly study first their patterns. This can help to optimize the use of available limited resources to enhance effective road safety and injury prevention and on the other hand sustainable transport systems. In particular, special attention is needed to regulate the time of traffic flows while considering the different needs of road users, transport modes and activities at certain locations, especially those much exposed to RTIs. Although by using the GIS generated map this study tried to visualize the prone-areas as low and high based on the RTIs they witnessed within the study period, there’s a vital need for in-depth future research to give a detailed profile of each identified RTI prone-area by using the most appropriate risk mapping or assessment tools. This requires a comprehensive study which covers Kampala city and Uganda as a whole, and other emerging cities elsewhere, especially in LMICs with rapidly increasing motorization and population growths.

**Abbreviations**

RTIs: Road Traffic Injuries; KMA: Kampala Metropolitan Area; LMICs: Low and Middle-Income Countries; arcGIS: Geographical Information Systems; UNCST: Uganda National Council of Science and Technology; UPF: Uganda Police Force; KCCA: Kampala Capital City Authority; KCBD: Kampala Central Business District; LOB: Light omnibus, OR: Odds ratio, LL: Lower limit, UL: Upper limit.
